# Social determinants of health and lung cancer surgery: a qualitative study

**DOI:** 10.3389/fpubh.2023.1285419

**Published:** 2023-10-31

**Authors:** Dede K. Teteh, Betty Ferrell, Oluwatimilehin Okunowo, Aidea Downie, Loretta Erhunmwunsee, Susanne B. Montgomery, Dan Raz, Rick Kittles, Jae Y. Kim, Virginia Sun

**Affiliations:** ^1^Department of Health Sciences, Crean College of Health and Behavioral Sciences, Chapman University, Orange, CA, United States; ^2^Division of Nursing Research and Education, Department of Population Sciences, City of Hope Comprehensive Cancer Center, Duarte, CA, United States; ^3^Division of Biostatistics, Department of Computational and Quantitative Medicine, Beckman Research Institute of City of Hope, Duarte, CA, United States; ^4^School of Public Health, Brown University, Providence, RI, United States; ^5^Department of Surgery, City of Hope Comprehensive Cancer Center, Duarte, CA, United States; ^6^School of Behavioral Health, Loma Linda University, Loma Linda, CA, United States; ^7^Department of Community Health and Preventive Medicine, Morehouse School of Medicine, Atlanta, GA, United States

**Keywords:** cancer, community support, lung cancer surgery, patients, oncology, quality of life, social determinants of health, structural determinants of health

## Abstract

**Introduction:**

Social determinants of health (SDOH) are non-clinical factors that may affect the outcomes of cancer patients. The purpose of this study was to describe the influence of SDOH factors on quality of life (QOL)-related outcomes for lung cancer surgery patients.

**Methods:**

Thirteen patients enrolled in a randomized trial of a dyadic self-management intervention were invited and agreed to participate in semi-structured key informant interviews at study completion (3 months post-discharge). A conventional content analysis approach was used to identify codes and themes that were derived from the interviews. Independent investigators coded the qualitative data, which were subsequently confirmed by a second group of independent investigators. Themes were finalized, and discrepancies were reviewed and resolved.

**Results:**

Six themes, each with several subthemes, emerged. Overall, most participants were knowledgeable about the concept of SDOH and perceived that provider awareness of SDOH information was important for the delivery of comprehensive care in surgery. Some participants described financial challenges during treatment that were exacerbated by their cancer diagnosis and resulted in stress and poor QOL. The perceived impact of education varied and included its importance in navigating the healthcare system, decision-making on health behaviors, and more economic mobility opportunities. Some participants experienced barriers to accessing healthcare due to insurance coverage, travel burden, and the fear of losing quality insurance coverage due to retirement. Neighborhood and built environment factors such as safety, air quality, access to green space, and other environmental factors were perceived as important to QOL. Social support through families/friends and spiritual/religious communities was perceived as important to postoperative recovery.

**Discussion:**

Among lung cancer surgery patients, SDOH factors can impact QOL and the patient’s survivorship journey. Importantly, SDOH should be assessed routinely to identify patients with unmet needs across the five domains. SDOH-driven interventions are needed to address these unmet needs and to improve the QOL and quality of care for lung cancer surgery patients.

## Introduction

Social determinants of health (SDOH) are factors that contribute to the conditions by which people live, work, age, play, and worship that determine their quality of life (QOL) and mortality (1, 2). SDOH are organized into five broad domains: economic stability, education access and quality, neighborhood and built environment, healthcare access and quality, and social and community context (3). While understudied in oncology research, SDOH factors impact the QOL of patients and their family caregivers (4).

Lung cancer is the leading cause of cancer deaths. Survival is low due to late diagnosis. Despite the proven effectiveness of screening for early detection, access is not equitably distributed (5). Patients also experience several detrimental health outcomes throughout their survivorship journeys that are compounded by SDOH factors (4). Non-clinical factors such as race/ethnicity, insurance status, education, neighborhood features, and income have been associated with perioperative complications and survival following surgery (6). Patients with private insurance are less likely to have postoperative complications (7) and disparities in postoperative mortality for non-white patients (8) from low median-income communities and lower educational attainment persist (7).

Additionally, patients commonly experience mental health challenges, such as worry about transportation, treatment cost, symptoms and side effects, lack of social support, anxiety about function decline, and impact on work (9, 10). As patients experience a decreased QOL due to the disease and treatment process (11, 12) (e.g., surgery and chemotherapy), social support (13, 14) and spiritual/religious wellness resources (15, 16) have been shown to improve outcomes. The importance of addressing psychosocial support access for patients is critical during their survivorship journey (17–20). Sex assigned at birth, age, and other sociodemographic factors have influenced supportive care needs (17). For instance, female status, poor emotional functioning, and younger age have been associated with increased use of psychosocial support services (19). With respect to QOL, prior research has also noted the importance of the provision of palliative care and end-of-life care support (21–23), including practices such as symptom management, education, and coping mechanisms for patients and family caregivers.

Furthermore, levels of economic stability can impact a patient’s lung cancer diagnosis (24–26), treatment access (27), and QOL (28, 29). Educational attainment may also inform delays in treatment referrals (30) and healthcare access for lung cancer patients (31). Finally, there remain significant challenges in neighborhood and built environment conditions, including occupational and residential exposures related to diagnosis and access to care (32–35). Neighborhood-level characteristics have been used to identify high-risk lung cancer behavioral patterns in Maryland (36). The negative effects of lower socioeconomic status on treatment and survival have also been determined with the effects of segregation and economic deprivation determining receipt of lung cancer surgery in Georgia (37). Thus, an individual’s geographical residence may determine their treatment and survival (36–38). However, the current literature on the impact of SDOH on lung cancer patient outcomes is limited and focused primarily on healthcare access and quality, economic stability, and social and community context domains (4). Research on lung cancer surgery patients is also lacking narratives from this population and warrants additional inquiry (6). Thus, to better understand potential barriers to QOL for lung cancer surgery patients, we explored SDOH-related outcomes across the five established SDOH domains.

## Materials and methods

### Intervention, sample, and setting

This study is a part of a randomized trial of a multimedia self-management intervention for lung cancer surgery patients and family caregivers from a National Cancer Institute designated comprehensive cancer center in Southern California.

### Procedures

Participants enrolled in the parent study were eligible for the qualitative study at 3 months post-discharge and following completion of the parent study. During informed consent of the parent study (before surgery), participants were able to select whether they were willing to be contacted for participation in the qualitative study. The parent study followed participants for up to 3 months post-discharge from surgery. Thus, many participants were in the post-treatment survivorship trajectory or completing additional adjuvant treatments based on the stage of the disease. A nurse interventionist and research assistant from the parent study invited participants who agreed to be contacted for the qualitative study. Patient eligibility criteria included: (a) diagnosis of lung cancer as determined by surgeons; (b) underwent curative intent surgery for lung cancer treatment; (c) a family caregiver (FCG) enrolled in the parent study; (d) age 21 years or older; and (e) able to read, speak, or understand English.

The lead author (DT) conducted the semi-structured key informant interviews (Appendix A) with a co-facilitator (*VS*). The interview guide was developed in collaboration with co-authors and pilot-tested with patients in the same data collection pool. Questions were developed in three-phases. To introduce the concept of SDOH to participants, Phase 1 included a review of the “A Tale of two Zip Codes” (Two Zip Codes) video (39) followed by three awareness questions. The Two Zip Codes video was developed by the California Endowment to detail the SDOH impact on life expectancy in the United States in the context of racial and economic discrimination. The video highlights these SDOH factors on health by comparing two individuals from affluent and disadvantaged communities. Phase 2 included questions on QOL and survivorship by SDOH domains, and Phase 3 questions were developed to solicit how SDOH information can be incorporated into patients’ survivorship care planning. Interview questions were developed based on previous research findings (4) and subject matter expert recommendations from our research team. The interview guide was revised and refined based on pilot test implementation.

Each interview lasted approximately 60 min and was conducted *via* Microsoft Teams to minimize travel burden for participants. Instructions on how to operate Teams were provided *via* email, and an outlook calendar invite was sent to participants before each interview. A nurse interventionist and research assistant also reminded patients *via* telephone and/or email and reviewed Teams’ instructions with patients before their scheduled interviews.

Demographic and SDOH information was obtained at the baseline of the parent study using the Protocol for Responding to and Assessing Patients’ Assets, Risks, and Experiences (PRAPARE) (40). This 21-item instrument measures SDOH domains for patients related to environment, economic stability, and social and emotional health factors. PRAPARE is a standardized patient and social risk assessment tool informed by research on SDOH and aligns with national initiatives (e.g., the Department of Health and Human Services’ Healthy People), federal reporting requirements, and the International Classification of Diseases-10 clinical coding system. Participants provided consent for this study during the parent study onboarding procedures. An institutional review board approved the study protocol and procedures.

### Data analysis

The conventional content analysis approach was used to identify themes from patients’ experiences (41). Codes and subsequent themes were derived from participants’ interviews and relevant research, or theory was used to interpret meaning from data. Our research team published a systematic literature review on the impact of SDOH on FCG as well as lung cancer patients which informed our interpretation of the data for this study (4). We used the US Department of Health and Human Services’ SDOH framework which includes five broad domains: economic stability, education access and quality, neighborhood and built environment, healthcare access and quality, and social and community context. The process included four independent coders (DT, VS, JK, and BF) followed by four independent reviewers (DT, VS, JK, and AB) who developed the initial content themes. Two reviewers (DT and VS) reviewed and finalized the themes. Coding and/or theme disagreements were discussed, refined, and resolved. Descriptive statistics were used to summarize patients’ demographic characteristics. For continuous variables, median and interquartile range were reported; sample size and proportion were reported for categorical variables.

## Results

A total of 106 participants of the parent study agreed to be contacted to participate in the qualitative study. Out of this total, we interviewed 13 lung cancer surgery patients for the study and were able to reach saturation with the sample size. Participants were primarily non-Hispanic (85%) and white or Caucasian (69%) with catholic religious affiliation (39%). Most patients (as shown in Table 1) spoke with their social support network five or more times a week (46%). Participants’ stress levels varied before surgery ranging from “very much” (15%) to “a little bit” (31%). English was the primary language spoken by respondents, and most individuals (85%) completed more than a high school education. Patients were either insured through Medicare (46%) or private insurance (54%) and had adequate access to healthcare services. In addition, respondents did not have any housing insecurities, transportation issues, or neighborhood safety challenges. While some patients were unemployed (17%) or retired (33%), most were working full-time (50%) and reported an annual household income greater than $100,000 (39%). Two patients were discharged from the Armed Forces and very few respondents lacked access to food, clothing, utilities, childcare, or a phone in the past year. One respondent did indicate a lack of access to “all utilities” and another participant did not provide additional information about utility needs.

**Table 1:** Lung cancer surgery patients’ demographic characteristics by social determinants of health (SDOH) domains (*N* = 13).

**SDOH domain**	**Characteristics**	**n (%)**
**Social and community context**	**Age** in years, Median (Q1–Q3)	67 (61–67)
	**Sex assigned at birth**	
	Female	7 (53.8)
	Male	6 (46.2)
	**Ethnicity**	
	Hispanic	1 (7.7)
	Non-Hispanic	11 (84.6)
	Choose not to answer	1 (7.7)
	**Race**	
	Asian	1 (7.7)
	Black/African American	1 (7.7)
	White or Caucasian	9 (69.2)
	Other^a^	1 (7.7)
	Choose not to answer	1 (7.7)
	**Religious affiliation**	
	Protestant^b^	2 (15.4)
	Catholic	5 (38.5)
	Jewish	1 (7.7)
	Other	3 (23.1)
	No religious affiliation	2 (15.4)
	**Household size**	
	Less than two	7 (53.8)
	Two	2 (15.4)
	More than two	4 (30.8)
	**Social support communication**	
	1 or 2 times a week	5 (38.5)
	3 to 5 times a week	1 (7.7)
	5 or more times a week	6 (46.2)
	Choose not to answer	1 (7.7)
	**Stress levels**	
	Not at all	2 (15.4)
	A little bit	4 (30.8)
	Somewhat	2 (15.4)
	Quite a bit	3 (23.1)
	Very much	2 (15.4)
	**Imprisonment in the past year**	
	Yes	0 (0.0)
	No	13 (100.0)
	**Refugee status**	
	Yes	0 (0.0)
	No	13 (100.0)
	**Domestic violence**	
	Yes	0 (0.0)
	No	12 (92.3)
	No partner in the past year	1 (7.7)
**Education access and quality**	**Language**	
	English	13 (100.0)
	Other than English	0 (0.0)
	Choose not to answer	0 (0.0)
	**Education**	
	< High school degree	0 (0.0)
	High school diploma or GED	2 (15.4)
	More than high school	11 (84.6)
	Choose not to answer	0 (0.0)
**Healthcare access and quality**	**Main insurance**	
	Medicaid/CHIP Medicaid	0 (0.0)
	Medicare	6 (46.2)
	Private Insurance	7 (53.8)
	Other Public Insurance	0 (0.0)
	**Lack of access to medicine or healthcare**	
	Yes	2 (15.4)
	No	11 (84.6)
**Neighborhood and built environment**	**Housing Situation**	
	I have housing	13 (100.0)
	I do not have housing	0 (0.0)
	Choose not to answer	0 (0.0)
	**Worry about losing housing (*n* = 12)**	
	Yes	0 (0.0)
	No	12 (100.0)
	Choose not to answer	0 (0.0)
	**Transportation access consequences**	
	Medical appointments or medications	0 (0.0)
	Non-medical meetings, etc.	0 (0.0)
	No	13 (100.0)
	Choose not to answer	0 (0.0)
	**Physical and emotional safety**	
	Yes	12 (92.3)
	No	0 (0.0)
	Unsure	1 (7.7)
**Economic stability**	**Employment status**	
	Unemployed	2 (16.7)
	Full-time	6 (50.0)
	Unemployed but not seeking	4 (33.3)
	**Annual household income**	
	$15,000 to $30,000	1 (7.7)
	$50,001 to $75,000	4 (30.8)
	$75,001 to $100,000	3 (23.1)
	Greater than $100,000	5 (38.5)
	**Migrant farm worker**	
	Yes	0 (0.0)
	No	12 (92.3)
	Choose not to answer	1 (7.7)
	**Discharged from armed forces (*n* = 12)**	
	Yes	2 (16.7)
	No	10 (83.3)
	**Lack of access to food**	
	Yes	2 (15.4)
	No	11 (84.6)
	**Lack of access to clothing**	
	Yes	2 (15.4)
	No	11 (84.6)
	**Lack of access to utilities**	
	Yes	2 (15.4)
	No	11 (84.6)
	**Lack of access to childcare (*n* = 11)**	
	Yes	2 (18.2)
	No	9 (81.8)
	**Lack of access to phone**	
	Yes	2 (15.4)
	No	11 (84.6)

^a^Other response denoted as “Hispanic”. ^b^Protestant religious affiliation included Baptist, Methodist, Lutheran, Episcopalian, Evangelical, etc.

### Theme 1: knowledge about SDOH factors, quality of life outcomes, and potential health impacts

Patients were knowledgeable about the concept of SDOH before watching the “Tale of two Zip Codes” video illustration. The video as a result reinforced concepts about the impacts of social and environmental factors on QOL (five subthemes). The discussion of socioeconomic privilege, access to parks, nutrition, healthcare access, and race/ethnicity were highlighted as determinants of positive health outcomes (see Table 2). There was also consensus on the usefulness of providers knowing SDOH factors to tailor the survivorship care plan of surgery patients (two subthemes). SDOH was seen as an implementation of a whole-person care plan that exemplified the attributes of a caring provider. Some considered the need for knowledge about resource availability before and after treatment, healthcare access related to affordability of co-payments and insurance, and a better understanding of the patient’s worldview to tailor health solutions. Patients did not recall discussing the impact of SDOH on their health outcomes with their providers before surgery. Patients also stated that while the information may have been useful, the priority of the provider was to treat their disease.

**Table 2:** Knowledge about social determinants of health impact on lung cancer surgery patients.

**Themes**	**Example quotes**
**Knowledge about SDOH factors and quality of life outcomes:**	
Socioeconomic privilege	“Well, I guess it’s kind of in a way, states the obvious. If you are from a higher socioeconomic background, you probably have better health outcomes, in general, then somebody in a poor neighborhood.”
Availability and accessibility of parks, walkability	“Well, I’ve worked in human resources for years, so I know how it works. So, I understand it very well. Small communities that are in the poor range versus the other ones. You can see that in well, we live in Southern California and South County has beautiful parks and you know accessible to walking and riding your bike where the metropolitan older cities do not. They’re trying. They’re getting better, but they do not.”
Determinants of health outcomes	“Yes, actually, I do not think I heard it put that way, but I have heard that where you live matters, and it contributes to your health outcome. Well, I know that I’ve had access to healthcare, and I know a lot of people do not, and I think that I feel that that’s probably related to economics. And I do know that genes do make a difference and also a young life, and they did not really talk about that too much. Or I guess that’s my understanding. I know that I feel that since I had lung cancer that may be living in LA is more polluted than, say, Denver. And so that could contribute to my health outcome. I guess that’s about it.”
Race/ethnicity related factors	“Well, I’m African American, so even if I had not heard it, I knew it. Yeah, absolutely. What resonated [with] me [inaudible] these kinds of discussion, that kind of information is so important for making not just this country, but the whole world, that it’s almost like so many people are even unaware of being involved in these kinds of discussions. Well, the community that we live in is a combination of both.”
Healthcare access and quality	“How do these people do it when you are sick and I’m sitting there in the lobby of [a hospital], watching these other major cancer patients and their journey and what they have to go through. And if you do not have health care? You’re and you are basically totally messed up because how are you going to [inaudible] even feel that you are getting the right quality of service. If you feel that you do not have any insurance. And if I did not have health care? I do not. I would have to sell my house to pay for my medical bills.”
**Effectiveness of including SDOH in survivorship care plan:**	
Impact of SDOH on QOL should be discussed before surgery	“So other people, I think it would have done them better, more beneficial. Because people aren’t optimistic there. They always think the other foots [is going to] drop and everything [is going to] be bad. So, I think if they would have known or they would have given you more information before the surgery, I think that would have helped. I do not know from my experience that they do offer. I mean, if they do talk about it, my physician never did. So, I think that it would be wise for them to have, to offer that information. Sorry, some people want it. Some people need it. And some people do not. But I think it should be talked about Prior to surgery.”
	“I think so. Because you they got to know. Do you have the insurance? Do you have the resources? Can you afford the copayments? Yes, I think that would be good for the treatment team to know. If someone needs additional resources, do they have the support behind them? Are they able to take care of themselves? Because some people get really sick and cannot get out of bed and others, like I was pretty much okay [and] was just tired.”
	“Oh yeah, no, I think, yeah, I do. I do. Because you know, I have been in sales for you know, 20–30 years or whatever and you know, knowing who your customer is exactly and what their kind of worldview might look like and the way they are looking at things certainly helps me tailor solutions for them that will fit. So yeah, I would think that this is helpful for caregivers and medical professionals, I would think.”
	“Yes. Well, I think if your providers are aware of your [inaudible], and if they care, right? So that’s a big part of it is whether you have a good provider that is a caring provider and wants to see you as a person, wants to treat the whole you then great. But sometimes I think a lot of times we are just another patient that walks in the door and you got 15 min and then I gotta get you out of here.”
Priority of providers was surgery	“So, it’s a hard question because the team did what they had to do and answered all my questions. Everybody’s not going to be the same [inaudible]. [It] depends on them and say, look, this is your condition, this is where [what we are] going to do. And they kept asking me, do you have any other questions? Do you have any other questions? But if you do not, ask them, you cannot [receive an] answer.”
	“I’m sure yes, I have no problem with that. It may have been helpful, but I do not know that it would have made a big difference, but I would not have a problem with them knowing about it beforehand. I do not know. I do not know. Things could have been changed. The surgeries were what they were and pretty much had to follow its course, so I do not think it would have made any a big difference.”

### Theme 2: economic stability challenges and financial toxicity-related concerns

Several patients experienced economic challenges during their treatment resulting in detrimental financial toxicity-related QOL concerns as described in two primary themes (as shown in Table 3). One patient returned to work to have access to health insurance and paid time off during treatment. Another patient’s worries about continuing treatment after the expiration of her Consolidated Omnibus Budget Reconciliation Act (COBRA) insurance benefits were a source of chronic stress. For others, financial insecurity stressors had always been persistent but were now exasperated by a cancer diagnosis, which was the case for a single mom with worries of not having sufficient savings for retirement. Fortunately, some patients did not have economic stability concerns despite high deductibles, and out-of-pocket healthcare costs associated with their treatments.

**Table 3:** Economic stability challenges and financial toxicity related concerns of lung cancer surgery patients.

**Themes**	**Example quotes**
**Health insurance access and financial insecurity**	“My cobra is going to end in August, so I’m looking to see how should [I] manage that. Because this chemo supposed to be 3 years, [and] chemo is pretty expensive per month. So, I have to wait, does not make sense back then, to even start it because I may not be able to finish it. So, there’s a lot of stressors. One day at a time… Well, the thing is I read something about the financial department [on] one piece of paper. Says well, you have to qualify financially, you have to have this income that income, but see, that’s not what I’m looking for. I’m looking for someone to guide me so that it will allow me, to be able to continue what I have now. So that I can have [hospital name] be able to do a specialty drug, and not have a high out of pocket expense for every doctor’s visit. You know, there’s a lot of things to think about. I mean, this is just one, one part of [my] life that I have to worry about, I have other aspects of my life that I have to worry about as well with my parents passing away. You know, so I have to get that squared away. So that’s a lot of my shoulders right now, you know.”
**No economic challenges or concerns despite the high cost of healthcare**	“I mean, it costs a lot of their money, but I mean. I’ll be paying for it for many, many years, but it is what it is. Yeah, sure. I mean, but I have high deductibles, I’m self-employed. So, I do not have a great coverage. I’ve got okay coverage and I think so far out of pocket, I have a $8,200 out of pocket expenses. Unfortunately, I had surgery in December. Which was $8,200 out of pocket. And then I started treatment in March, which is $8,200 out of pocket. So, you know, we are talking, you know, [over $10,000] just right off the top. Yup, that’s a big chunk of change that I did not have sitting there. But again, it is what it is and I’m blessed to be able to pay my bills. You know, there’s a lot of people out there that cannot even afford to pay [their] bills or get the treatments or whatever. I’ve never known hunger and I certainly was not pushed to hunger by the treatments.”
	“I always have financial [security] at the back of my mind, you know, being a single mom and put one kid through flight school and other one through bachelor’s degree, there was just a lot. And so now it’s time to start saving, but I already should have been retired, right? So, in my mind, there’s [an] economic [insecurity] as far as financial stability, I do not feel like I have a lot of it, but I’m working towards it. Other than that, I had no insecurities.”
	“My boss had laid me off. But financially, I mean, we are okay. I’ve probably made like somewhere around 80,000 a year for what a while. I’m on Social Security. I’m not working. My wife works. She works at [a hospital]. We do not own our home. You know, we did own a home [inaudible]. I was working too much to find another one [home], so we rent. But we have a nice house. The townhouse, I got a good landlord, and then we do not have any particular stress that way. Not living high on the hog or anything, you know, and I do [have to] watch my spending. But I think we are set up okay, we are we can keep going until my wife and I die without having to worry about [money].”

### Theme 3: impact and importance of education access on QOL

Four subthemes described the variable experiences of education on QOL for patients. They included the importance of education for healthcare navigation, lifestyle decision-making strategies for health behavior change, the connection among education, economic mobility opportunities, and better health outcomes, as well as no impact of education on QOL (as shown in Table 4). The benefits of matriculating through an academic degree program were described by participants to include the ability to conduct personal research, comprehension of medical terms or increased health literacy, and decision-making strategies related to the types of questions to ask providers to navigate the healthcare system. There were also benefits for some that propelled lifestyle decision-making strategies or modifications of unhealthy behavior patterns. However, as described by a few participants, the knowledge of a topic does not always result in avoidance of actions such as smoking that have known detrimental health consequences. In addition, participants also briefly discussed the connection between education and economic mobility opportunities that could lead to better health and improved QOL. However, most participants did not agree with the statement “*people with higher levels of education live healthier and longer lives.”* Their understanding of education extended beyond the mere attainment of an academic degree. Several patients attributed their health literacy to their lived experiences, which in turn resulted in their improved ability to navigate their survivorship journeys and better overall health. They also noted that having a positive and/or inquisitive mindset and professional background (e.g., real estate and claims adjuster) had a positive impact on health outcomes.

**Table 4:** Education quality and access impact on quality of life for lung cancer surgery patients.

**Themes**	**Example quotes**
**Importance of education for healthcare navigation**	“You learn your basics, but nowadays I think education plays a big part of everybody’s lives, and I just think if they know more about healthcare and where they are going, I think it’s great. I because of my dad going through cancer, every time a doctor would ask me a question, I was there Googling it. OK, what does that mean? …So, you become an advocate of your own parents. Now with mine, obviously, when they told me I had a mass, I am Googling what is a mass mean…So, I think if you have the right education, and you know how to ask the right questions. I’ve learned that the more questions you ask, the better you are gonna be with yourself.”
	“Yeah, if you have education then you know, just through memory, muscle memory… People who go to college, they know how to take notes. They know how to do research because you have to do research paper, right? So yeah, that’s what we have to do is we have to research what cancer is the type of cancer you have? What’s good for this particular type of cancer? So yeah, comes into hand, comes handy to have higher education.”
	“[I] have a bachelors in chemical engineering and I did postgraduate work in business. Yeah, I think so, because I am technically trained and I could read some of the medical reports and I could ask, you know, good questions of the doctor. What does this mean and what does that mean? So, I’m not flying blind. Like, maybe most patients would be.”
	“I see doctors a lot more often now. Before I barely went to the doctor. I had to be really sick before I would go. You know I’m learning a lot about it, you know, when they mentioned something new, I read up on it.”
**Lifestyle decision-making strategies for health behavior change**	“Oh yeah, I agree. Well, if you are educated, that means you probably read, you are aware of developments you learn over time which behavior to avoid, which behaviors do [you] accentuate for healthy living. You know, I’ve been a jogger, I walk, and I do not overindulge. Never had drugs in my life. That kind of goes along with that, I think.”
	“Yes. Well, you know, I’m not personal in this area by any stretch of the imagination. But you know, I tried to be for a number of years now, you know, we have tried to be as healthy as you can and find out as much as we can about the things that do affect our health. You know outside of our own body and so, I’m always looking for information that can make you know they can keep me more knowledgeable about what’s the best things to do. The best way to live. They keep me as healthy as possible.”
	“Because I, you know, I’m college educated. And you know, I think I’ve lived a pretty good life and I’ve tried to do reasonably healthy things, you know, since I’ve learned how important that is. The only thing I can think of is the fact that I did smoke all those years and I’m assuming that even though some people get cancer, that mine was a well, you know what mine was. So, I’m assuming that my [actions] has something to do with that because Cancer is so prevalent among…, so much more prevalent among people who smoke than those who do not.”
**Connection between education, economic mobility opportunities and better health outcomes**	“Oh, I think that yes, I would agree with that in a general sense. I think that stress plays a big part, so sometimes that can counteract that. But because somebody’s educated, can probably kinda think for themselves on how to get their needs met. That’s what I’m thinking, and I guess that’s all I have to say on that… We’ll make more money also, someone who’s more [educated] make more money and then therefore they can maybe have better health care that way. I have a graduate degree, but I went back to school later on, so I got my bachelor’s degree and master’s degree. I graduated in 2015. So, before that I just had some Community College education.”
	“I think it’s true because people [who] are more educated, usually have better jobs, have more money. But then it’s not true with my case because I dropped out at 9th grade. And then in my 30s, I would get my GED. But then this what I’m working as a claims adjuster. I just kind of fell into it, but I’m making about 90,000 a year. I probably would be in the different field, maybe making more money or it could have affected it. You know, I could have been educated and got into a different field and made more money. I would have the resources to eat better, exercise, be active. I think it’s a little different because you can have those degrees nowadays and not be able to find a job. So, you gotta be careful on what you are educated [in] and if there’s employment in that area. Oh, if they are taught how to research, you know and study up on their diagnosis and treatment and people that usually go to school, you know, higher education, they are from wealthier families. So, they are upbringing is different than you know, I was raised by a single mother who basically made minimum wage, so there wasn’t much money in the family growing up.”
**No impact of education on quality of life**	“My education, I mean, I never finished high school. I’ve been to, I do not know how many colleges and stuff like that, but you know, I did not even finish high school. But it did not seem to stop me for anything. I do not know. I do not think it affected anything. I think it’s more of a mindset, you know. It’s kind of this, you know, I do not have any fear of it.”
	“Well, not necessarily a degree, but the person, if they are well read and study on their own. The information is available, so they have got an inquiring mind, access to the internet. It’s not strictly related to a degree. If you have a degree, you are more likely to access those tools. But anyone, even without a high school diploma, if they are inquiring and curious, the information should be out there.”
	“I do not know, I do not think, I mean I’ve got a master’s degree. I got a business and MBA, but I do not know. I mean, I guess you know, I’m 56 years old, so I [went] to school at a time where they still put projectors [inaudible]. So maybe it helped me, you know. But I do not know if they had a direct, I do not know that I can directly draw a line between like what happened here [and my education level]. You know what I mean? My wife, though however, I should say this, my wife is a speech pathologist. So you know, and obviously she’s not a doctor, not an MD, not you know, but you know works in the healthcare field, works with doctors, understands how to read clinical studies, knows the insurance game you know and all that sort of stuff so that and offer education and having her as a resource to me, now that was a big help.”
	“I think I agree for the most part because that’s with an assumption, that the assumption is that higher educated people are gonna try to get more information. And as it pertains to health, you know that means that, that person most likely is going to try to get more information than a person who is not educated right? Yeah, but I’m not sure that has anything to do with my education, to be honest, which I think that’s just the kind of person that I am. Yeah, I’ve been a curious person in my whole life.”

### Theme 4: access to quality healthcare including insurance status, lack of follow-up after surgery, and COVID-19 challenges

Seven subthemes described participants’ experiences with access and receipt of quality healthcare (see Table 5 for details). Overall, access to healthcare was not a major problem for participants. Positive aspects of healthcare access were common, with overall satisfaction with the quality of care. Proactive postoperative follow-up by the healthcare team on postoperative symptoms and overall wellbeing was viewed as quality care. A quality care environment that promoted clinical excellence, safety, and compassion was important for participants.

**Table 5:** Access to quality healthcare included insurance status, lack of follow-up after surgery, and COVID-19 challenges.

**Themes**	**Example quotes**
**Access to healthcare:**	
Challenges with access	“For me, the health care HMO system is horrible. I was so frustrated and pissed off. I mean, I was getting pretty aggressive, but I mean not like, you know, hurt somebody or something like that, but I definitely wasn’t backing down or accepting anything any of the doctors. I mean, I do not have the greatest review of doctors at this point in my life. You know, they also tell me such so much bullshit. It’s unbelievable. But so, I changed to PPO, and I did this so I can get into City of Hope also.”
	“I have PPO HMO and I pay. A lot to have that which is kind of sad that people do not have that flexibility, do that. So I know that I was treated extremely well. I see myself and I go. What if you do not have this kind of health care?”
	“And I scary because I’m almost. I’m almost at the retirement age and this is what comes through my mind. Ohh my gosh, how am I gonna keep up this this insurance? You know, because as you get older, things happen. And how have you thought about how you are going to keep up with your insurance after you retire?”
Positive aspects of healthcare access	“I have come to rely on my health care providers and to follow direction. I think within the last certainly 10 years I’ve had a shift where before that I thought I’m in charge of myself and if I need to go to see the doctor, I’m gonna listen to what they say. But I’m gonna decide for myself. I’m a lot more willing just to okay, I just have to trust this person. They know more than I do. You know, it’s no one’s ever 100% right. But I might as well follow their direction and I can let go of that responsibility. Right. I do not, you know. And actually, that’s been a little freeing.”
	“I know that after my surgery they certainly were concerned with my comfort and how I was doing, you know. I’ll say mentally, just kind of generally they were very inquisitive and kind and wanting to make sure I was handling things well. And even when I was home and I did start to have pain, some nerve pain, that was really great how I they were so much available for me to call in, talk to the nurse, they connected with my doctor’s office.”
	“They had a little stuffed bear with a shirt that was called [inaudible]. And so of course I used him to cough. But I was really glad he was in the room. I wasn’t able to have either my daughter or my boyfriend comes in because they had both been exposed to somebody who had COVID, so I was there alone. I have my little bear and I love that. You know, the simplest little thing.”
Palliative care access and knowledge	“I’m aware of it, but I did not think it was pertinent to me I guess, I think of it as people in Hospice or whatever, so it’s probably a broader term than that [knowledge about palliative care services, and no one mentioned the service]. With the things they were doing ongoing and I was wasn’t aware of. That would have fallen into that category. Mainly in the symptoms, I think particularly the cough. There’s something I would actually probably use some help on and the shortness of breath the more concerned long term is this what I’m gonna be dealing with or will it get better over time?”
	“I guess that was aware they existed, but I’m concerned like I think I’ve done pretty well. I have, you know, it’s palliative care sounds like the last hope or resistance or so. No, I’ve had no need for those, thankfully.”
**Description of survivorship journey challenges:**	
Symptoms	“When I got out, I mean, you know, it’s hard to breathe…. Well, my little weird sensations, little pains or numbness or whatever it is are in the front…. And you know, my whole thing was at that time was the cough. You know, I was coughing constantly… the coughing was driving me nuts. I was like around the clock…. You know, sleep is just it’s hard to get it to sleep…. Being constipated is a real frustrating experience and then does that to you.”
	“I think I was really hard on myself. I just did not like the uncomfortable of not being able to breathe right sometimes. I kept I had a panic attack once, and I just thought ohh my gosh, I cannot breathe. I cannot breathe and I had to tone myself down and put myself in a space of. Just a. Of a different realm. Thinking about being in the beach like your background is and just calming myself down so I could breathe again normally. The pain was a little uncomfortable only in my sense, and then, you know, you are a woman. So, you wanna look your best. And the idea that I could not wear a bra for a while was kind of comfortable. So I had to wear all this stuff on top of it. So. But then it just got better. I just realized you are your worst enemy.”
Recovery	“You know, I still wanna get stronger. I still want to gain some weight. I mean, I’m not giving up there. I went out with normal life, so I can golf or do whatever I feel like doing. I’m still real weak and I have not got all my strength back. But you know, I’m getting a lot stronger and that’s a slow process. That’s the other part. Healing is a slow process.”
	“I went back to work three days after surgery, and I can only put in 4 h a day. Now I’m putting in 11 h a day. But I’m able to.”
	“Well, I’m still getting used to it. I’m 56 I’m a nonsmoker. I’ve been really healthy. My whole life I’ve invested a lot in my health and having this happen to me was you know, like a six-month slow-motion airplane crash. So, I feel like. I feel like my life is potentially shortened. I feel much more unsure about the future then I used to be. That could be a reason God gave us to me. I do not know. I’m not very good at present. What else can I say? It sucks. I do not like it at all. I mean, I’m not happy with it at all. I’m still, probably going through all the stages of grief, you know, in regard to it, and I really do not know how I’m supposed to manage the intensity and level of work that I’ve done in the past professionally and do all the things that I’m doing now to try to keep me from getting cancer again in the future, so I have no clue. I really do not know how that’s going to work out.”
	“As much as I think I’m strong, I do not have a lot of leeway left for other things, I think, that I had before.”
	“But you know, I have to do the screenings cause I guess there’s still a 50% chance that it could come back. So that bothers me a little bit. That’s essentially the extent I think I’m still processing the fact that I’ve gotten this diagnosis.”
Care Coordination	“Before I did not have that much knowledge so that that part. Like I think I needed a flow chart. I needed to know what each doctor was there for me. Because you can say one word and say hey, so what is that doctor do really for me? So that’s just me though, because I did not. Just because this doctor had a title, I did not know what he was supposed to do for me like, why am I seeing you? Ohh Okay, it’s because of this Okay. Well, a surgeon? Yeah, that’s pretty obvious. I know what a surgeon does. But I did not know what a Pulmonary doctor was. But yeah, I just I guess maybe if you are in shock. You’re still receiving the information.”
**Negative experience with healthcare system:**	
Lack of follow-up after surgery	“The only thing that had me very, very upset was I had surgery on a Monday. They took 15% of my lung out. I was just discharged on a Wednesday. And not one soul, not one soul called me at all the next day, the second day, the whole weekend. There was one girl. She’s on the research team. She’s the only one that called on the following Tuesday. That’s it. Not a nurse. Not the doctor’s office. Nobody. And I was kind of a little I’m gonna tell you I was ticked off…”
Delays in care	“But for 10 years I told him I cannot breathe. I cannot breathe. I cannot breathe. And he kept telling me, I do not know what to tell you. I do not know what to tell you. I do not know what to tell you. I’ve done X rays of your lungs are fine, but really, I had COPD that wasn’t being treated for 10 whole years... So I went from nothing wrong with you, to you have already died practically…And I truly believe if they had caught it sooner at least started treating it sooner. It would not be to the degree that it is.”
**Positive experiences with healthcare system:**	
Healthcare team	“They knew where they were [doing]. They never lost sight and they just followed me through it.”
	“You do the whole thing by yourself, so they become your family. And yeah, they just make you. You’re just so grateful for all these people that are working. You know, through the holidays, it does not matter what the weather is like, it just and everybody. I never met anybody there that did not love, love their job.”
Care environment	“You’re with the right people. There are people that support you as people that are going through what you are going through. The whole environment was just made you feel like you are in a world class place and that you are safe. Or at least you are in the best place you could be…”
	“I do not have enough good things to say about that because one of the things that’s really important is that I can go there in the morning and I can have my scans and see him a couple hours later and walk out of there knowing that my skin was clear. That’s huge. As opposed to getting a scan done here in [hospital name] and then make an appointment…”
**Burden of traveling long distance to obtain care:**	
Challenges with family and friends visiting after surgery	“Yeah, cause none of my friends could come up visit me because it’s 50 miles away. When I was in the hospital for three days. Cause gas. It’s not because of you guys. It’s not because of the distances cause of how much it costs anymore right now.”
Challenges with treatment	“I mean, it’s possible if there’s no traffic to get there in 45 min. That’s also possible for it to take an hour and a half. Hello, it’s a hassle, you know. Especially now with gas, I mean gas is so expensive. It’s like oh no. You know that’s good. Take a big chunk out to drive there.”
	“…where I got my treatment is 2 1/2 h from here. And Lancaster, where I got my chemo treatments, is about an hour and a half. It was not convenient, but we do have a Cancer Center here. They do not have a thoracic surgeon and they do not do lung cancer here. When you leave chemo, you do not usually feel very good and then just spend a couple hours in the car, usually is not great.”
	“So, if my surgery was on Monday, I had to go up on Friday to get my COVID test. And then Monday I came in for surgery. Not that I would not have done it. It’s just I’m thinking you have offices down the road. Why cannot it just go there? But you cannot? Not yet.”
	“…42 miles. I would like to walk next door if I could, you know. Yeah. But you know what? I if I have a choice between great care Versus going next door, I would take great care every time if I have to. I have, you know, we have gone out of state for things. No. You know, really in Southern California and, you know, things can be a way away. And so, you know, we used to drive and then I’m sure everyone wants to be able to do what’s the most convenient thing to do, you know…”
**Delays in lung cancer diagnosis due to COVID-19**	“First of all, there was COVID last August, September, October COVID was very, very high. I he could not find a pulmonary specialist that could take me. That was number one because they were dealing with COVID patients in the hospitals. They were had no availability to get me in…”
	“…nobody is doing biopsies, nobody. I mean nobody…And so at this point I am so upset. I figure I’m going to die any hour. I mean, I did not know how sick I was, I did not know anything really…”

Others shared examples of challenges in accessing healthcare that were associated with insurance coverage. Despite having insurance coverage, access to quality healthcare was not guaranteed for many participants. Fears and anxiety around insurance coverage as participants faced retirement age were prominent. Negative experiences with the healthcare system included a lack of follow-up after surgery and initial delays in diagnosis. Due to COVID-19, participants described delays with initial diagnosis due to the inability to see specialists and have biopsies. The authors described the impact of the COVID-19 pandemic on care delivery and QOL for this population in a previous publication (42). Additionally, travel burdens to obtain cancer care and for family/friends to visit were primarily financial, with high gas costs. For participants, long trips after chemotherapy were challenging due to post-infusion toxicities.

Finally, descriptions of survivorship journey challenges were mainly focused on symptoms, recovery, and care coordination. Participants described challenges with dyspnea, prolonged coughing, pain, constipation, and weight loss. They described recovery and healing as a slow process, and frustrations with their inability to participate in activities that they used to enjoy and return to work. On the survivorship journey, participants were still “processing their diagnosis” and “grieving” the reality of being diagnosed. Challenges related to care coordination included not knowing which clinician was responsible for different aspects of their care.

### Theme 5: neighborhood and built environment disparities and health impact variability

The overarching theme that emerged from the patient interviews was a common understanding of significant disparities in the neighborhood and the built environment, even among those participants who were not negatively affected by these factors. Four sub-themes centered around safety, air quality, access to parks and green space, and other environmental causes of cancer (Table 6).

**Table 6:** Neighborhood and built environment disparities and health impact variability for lung cancer surgery patients.

**Themes**	**Example quotes**
**Safety**	“I live in a town now that is fairly mixed where you can be in a very wealthy kind of enclave and then go a few blocks and be in a very poor couple of blocks... on my street, I feel very safe... but about a half a mile away, there’s been shootings and whatnot.”
**Green space availability**	“I’m about 2 1/2 miles from a popular hiking trail... I can bike there and it’s a beautiful stream with trees.”
**Air quality**	“Air pollution because I never smoked. And so of course we do not know the exact cause, but I suspect maybe just pollution in general.” “I live in Southern California and Southern California is noted for having bad air quality, for example. But I would have paid attention to the area that I live... It would have made me aware of things so that I could make sure that the choices that I made would be more beneficial for me and my family from a health point.”
**Other environmental exposures**	“I was exposed over the years to certain environmental effects by being in large factories.”

Although most participants felt that they lived in relatively safe areas, they acknowledged the stark disparities between safe neighborhoods and unsafe neighborhoods. Some participants noted that safe neighborhoods were sometimes geographically very close to unsafe neighborhoods. All the participants in the study lived in Southern California, so it was unsurprising that air quality was a common issue. Several participants felt that poor air quality contributes to developing lung cancer, particularly among never smokers. Other participants commented that certain parts of Southern California have better air quality—communities close to the beach and some less densely populated areas. Participants noted very tangible air pollution, which they could sense from traffic or recent wildfires. Generally, participants felt more affluent areas have better air quality.

Participants also commonly expressed they had adequate access to parks or other green spaces in the form of hiking trails. Some patients noted that local parks were not well maintained. Others commented that although they had parks in their neighborhood, they seldom used them. Finally, participants raised the issue of other environmental exposures to carcinogens. One participant wondered whether living near a gas station may have affected her health. Others questioned whether prior experiences of living near factories or other industrial complexes may have caused their cancers.

### Theme 6: social, interpersonal, coping, and community context perspectives

Participants provided perspectives about their emotional, relational, and coping strategies for dealing with the challenges of their survivorship journeys including access to familial, social, spiritual, and religious support systems. As shown in Table 7, two main themes and four subthemes describe the experiences of patients. Overall, patients stressed the importance of having a positive outlook, with one patient noting that a person could be their own worst enemy in this process. Particularly, some patients discussed the stigma and self-blame of being former smokers, the consequences of their past actions that led to their diagnosis, and the burden on their families which weighed heavily on them.

**Table 7:** Social, interpersonal, coping, and community context perspectives of lung cancer surgery patients.

**Themes**	**Example quotes**
**Coping with diagnosis, treatment, and self-blame:**	
Stigma and self-blame	“Well, I’m Angry with myself a lot. Because I smoked all my life, and it could have been avoided. You know, it’s just one of those things. And I have a lot of anger and guilt that I put my family through this and things like that.”
	“I smoked up until I found out. And had for a long time and at that point I quit, and people say, well, how did you quit, and I go dude, when you see these people that are trying to save your life. And you really keep smoking. That just like does not even make sense. You’re just going to keep killing yourself. Well, all these people are trying to save you. And not ask for a thing back.”
Emotional impact of diagnosis	*“*I can say that you know, like, you know, I guess I was very unprepared for the emotional impact of cancer. And I do not really blame that on anybody, right. I do not blame that. And I cannot say that what I went through is like, whatever. I do not know what everybody else wanted. But I certainly was really thrown off by that.”
**Familial, social and spiritual support:**	
Social support	“You know, there were days that I did not want to walk. I just wanted to lay down and just…I just did not wanna do that and they would come around and goes. Come on, let us go do it [go for a walk]. Let us go do it. Half a block. Two blocks. Come on. Come on. So that helped. That really helped. So, I have an amazing support.”
Spiritual and religious support	“So, for sure you know, I come from a very Christian background, and I believe in the power of prayer. And I had, I was online. If I was on a lot of prayer chains and prayer lists and I know that has always [played] a role in particular, [my] own attitude. I believe that it can be the difference, honestly. Between how you handle something. I do not know. I think it just gives you a bit of confidence and a bit of peace that you are not ever…you know, I never felt alone. I never felt that I did not have someone to turn to. You know, I never felt like I was abandoned in any way. And I think that as a Christian that has a lot to do with that. That I never felt alone. Yep. I know, I know, a lot of people would not consider those exactly like a mutually friendly, but I do not know…right, right. Thank you, Jesus, for the Xanax.”
	“I think it’s more spirituality. My mother was a big reader of *Kahlil Gibran* who’s a prophet, and so we kind of read his books and we really enjoy him, and I wasn’t brought up really religious. I did bring my son up Catholic so and I do pray, and I am Christian and so I think all of its…I think all of that combined…”
Isolation during COVID-19 pandemic	“I think I probably could have done more. I could have talked to more of my close friends. COVID has kind of impacted me socially that way. I was so used to, I have a handful of good friends that we would go out to lunch, dropped breakfast, or go for a walk. And my friends typically were pretty COVID isolation, and I felt if I was outdoors, I was pretty comfortable getting together. But my friends were not necessarily so and so I’m not super good at talking on the phone. And so, I let that social network gets a too far away. I did connect once I got the diagnosis, I knew it was important for me to touch in and tell people what was going on, and I did that and there were a few phone calls but probably not as much of my friend network support, and that’s on me as much as on them.”

Patients used varying coping strategies to navigate their diagnosis and treatment journeys. Most patients were unprepared to deal with the emotional and physical ramifications of their diagnosis and treatments which impacted their ability to breathe, work, and socialize, resulting in decreasing their activity levels and minimizing their caregiving responsibilities for other family members. Several patients initially withdrew from their familial and social relationships but eventually found solace in allowing the care and presence of their social network to provide support. Many noted that their familial and social relationships were supportive and encouraged activities beneficial to their recovery that they would not have otherwise been motivated to complete. Participants who were dually patients and caregivers experienced stressors related to caring for children with special healthcare needs and other family members during their recovery. Others discussed feeling isolated or disconnected from relationships in part due to the COVID-19 pandemic.

In addition, spiritual and religious support was used by patients as a tool for connection, and a way to cope with their mortality using traditional and non-traditional religious formats. Patients’ actions ranged from lighting a candle before prayer, asking God for forgiveness, blessing of good health, going to church on Sunday in person or *via* Zoom, returning to the practices of Catholicism to receive their last rites, and reading poetic essays from *The Prophet* by Kahlil Gibran about love, life, religion, and death.

## Discussion

Lung cancer surgery patients experience an array of detrimental factors related to their survivorship journeys that are compounded by SDOH conditions. Despite most participants having health insurance, they experienced several challenges related to financial toxicity, access to quality healthcare, neighborhood and built environment accessibility and exposures, as well as social and interpersonal barriers due to their diagnosis and treatment. They were knowledgeable about the impact of SDOH on their QOL, and the potential effectiveness of including discussions of SDOH in their survivorship care plan. Patients, however, understood that the primary directive of their healthcare team was to treat their disease.

Financial toxicity is one of the most common concerns for patients with cancer including lung cancer (43). Similar to previous studies (44, 45), some of our participants experienced distress related to medical insurance status, out-of-pocket costs, and treatment expenses that negatively affected their QOL. Fortunately, most patients did not have economic stability challenges or financial toxicity concerns. According to Hazell et al., protective factors against financial toxicity for lung cancer patients include older age, white race, employment status, having Medicare insurance, and an annual household income of more than $100,000 (44). Patients in our study were from privileged socioeconomic backgrounds with annual household incomes greater than $100,000 and white, with Medicare or private insurance. For example, one patient spent over $10,000 out of pocket during the initiation of their lung cancer treatment, and another patient was on social security and receiving spousal financial support with no economic stability concerns. Although we lacked representation from minoritized and under-resourced communities who carry a great burden of the disease, it is critical to examine SDOH disparities (46, 47) including financial toxicity concerns and their impact on under-resourced and minoritized lung cancer surgery patients in future studies. Doing so, using mixed methods with the use of validated screening tools such as PRAPARE (40) or the comprehensive score for financial toxicity (48), may better our understanding of the impact of financial toxicity on QOL and survival.

Educational attainment is associated with economic mobility opportunities which influences other SDOH factors such as income, healthcare access, food and housing security, transportation, and neighborhood residence (1, 2). Participants in our study did not discuss challenges related to food, clothing, and utilities. English was the primary language, and most patients had more than a high school education. Education is associated with survival rates of patients with lung cancer (49, 50). Patients of higher education have better survival rates and earlier diagnosis of disease than patients from lower educational attainment (e.g., grade school education). Additionally, the definition of education for patients and its impact on their QOL extended beyond a formal academic degree. An individual’s lived experience provides similar health literacy skills as completing an academic degree. This speaks to the potential limitations of solely relying on questions such as “what is the highest level of school that you have finished?” (40) to determine the impact of education on QOL. While the literature on the positive association between education and longevity is clear (51), determinants of educational attainment for lung cancer surgery outcomes are developing. It is important to include parallel educational experiences in addition to academic degree attainment when determining the impact of these variables on QOL.

Furthermore, SDOH disparities in healthcare impact the quality of lung cancer surgical care, management, and survival of lung cancer patients (52, 53). These healthcare disparities are associated with less use of surgery, more frequent use of more invasive surgical approaches, and lower postoperative survival rates (52). Minoritized groups including Black, Hispanic, American Indian, and Alaskan Native Americans are less likely to receive surgery for early-stage lung cancers even when adjusting for socioeconomic variables (52). As discussed by Bonner and Wakeam (6), there is a “*de facto* segregation” of lung cancer surgical care where non-white individuals on Medicaid or uninsured are more likely to receive treatment at low-volume hospitals where the quality of care maybe compromised, which consequently may impact the short- and long-term survival and overall QOL of these patients. With an underrepresented sample of these groups in our sample, we lack the data to adequately determine the burden of SDOH factors on healthcare access and quality. However, the burden of access was present in our study from the context of future worries about receiving care due to insurance status (e.g., ending of COBRA). Additionally, as the literature on SDOH disparities in lung cancer surgical care continues to expand, future research designs could benefit from including variations in outcome measures including volume, specific clinical complications, long-term survival, and mortality (6). The evaluation of these outcomes in conjunction with increased inclusion of socioeconomic information may inform disparities-focused interventions that improve access and surgical care delivery for patients.

Participants also acknowledged the relationship between their neighborhood and built environment and health outcomes. Safety, green space, and air quality were determinants of better QOL for participants. Patients also questioned the carcinogenetic effects of environmental pollutants due to proximity to factories, gas stations, and other industrial complexes. Pizzo et al. found significant associations between environmental pollution from a sewage and industrial plant in Italy and increased lung cancer risk for individuals living within 1.5 km (54). Patients residing in lower socioeconomic neighborhoods in Southern California with higher levels of airborne pollutants (e.g., PM_2.5_ exposure) have an increased probability of having a *TP53*-mutated lung cancer diagnosis which is associated with poor survival rates (55). Similar findings were shown by Yu and colleagues, with an association between air pollution from the combustion of coal and aggressive tumor biology for Chinese residents (56). In concert with social factors, future research designs should incorporate biological and environmental assessments of patients to better understand the burden of SDOH on QOL.

Moreover, social and community factors can determine patients’ QOL throughout their survivorship journey (4). Factors related to their psychosocial wellbeing, community engagement, and social support availability contribute to their health (1). Participants in our study received social support from their family and friends as well as through their spiritual activities and religious networks. Social support is an important factor for improving QOL for lung cancer patients (57). Particularly, the availability of social support for lung cancer patients is important for symptom management and better psychological and physical QOL (58, 59). For instance, early implementation of an interdisciplinary social support care model had long-term QOL improvements related to psychological distress 12 months after lung cancer surgery (60).

Additionally, the largest religious affiliation of participants from the study was Catholic (38.5%), and few had no religious affiliation. We did not quantitatively assess the spiritual wellbeing of patients but explored their understanding of their religion or spiritual support through interviews. It is important to note descriptions for these terms as their assessments are not synonymous and some patients provided distinctions between their spiritual practices that did not include religious doctrines. Spirituality can be described as the belief in a greater energy or force beyond oneself and the actualization of that belief in connection with self, others, nature, and the sacred (4). Religion includes traditions, rituals, and social practices combined with the belief in an unseen world and a deity which is often represented through doctrines (61). Religion can be an expression of one’s spirituality and is not always dependent on a religious affiliation.

Nevertheless, spiritual support positively impacts perceptions of disease (62) and can be protective against emotional distress for lung cancer survivors (63). Spiritual wellbeing of these studies was assessed using the Functional Assessment of Chronic Illness Therapy (FACIT-Sp-12) which includes measurements of meaning and peace, and the role of faith in illness (64) to understand the protective factors against emotional distress of patients. A religious and spiritual support intervention delivered by chaplains also has demonstrated similar salutary effects on lung and gastrointestinal cancer patients’ QOL (65). Religious support for the intervention was used to measure religious involvement which included three dimensions of religiosity: organizational religious activity, non-organization religious activity, and intrinsic religiosity (66). Spiritual wellbeing was also measured using the FACIT-Sp-12 tool. The critical importance of social support including spiritual and religious support is well-established (57). However, the integration or formal assessments of these support systems for lung cancer surgery patients is not well-known for patients throughout their survivorship journeys. While our patients discussed the importance of these systems for their QOL, their needs were not assessed before their surgery. This begs the question, how do we integrate these SDOH assessments into healthcare practice and provide support when needs are identified? This question is out of the scope of the current study, but an important next step is answering this question for future interventions for this population. Additionally, we recommend that SDOH assessments be considered a fifth vital sign (67) for lung cancer surgery patients and embedded into the standard of care practice and workflow.

## Study limitations

The findings should be considered in the context of several strengths and limitations. This study used a mixed methods approach to access barriers to QOL of lung cancer surgery patients from an NCI-designated comprehensive cancer center in Southern California. The use of PRAPARE in conjunction with qualitative questions across the five broad SDOH domains provided an informative narrative on SDOH disparities for this population. The use of a multimedia tool “A tale of two Zip Codes” supported the discussion with participants on SDOH. The use of Microsoft Teams is a notable strength as the burden of travel was minimized and allowed the research team to pilot the teleconferencing technology for qualitative data collection. The main limitation of this study is the lack of representation from under-resourced and minoritized communities. However, the sociodemographic background of participants mirrors the catchment area of the cancer center. We also did not address perspectives from family caregivers or clinicians in this study, but these perspectives will be evaluated in future studies as both groups were involved with our data collection efforts.

## Conclusion

Lung cancer surgery patients experience several barriers during their survivorship journeys combined with SDOH influences that impact their QOL. Notably, some patients experienced financial toxicity but reported that they received quality healthcare and social support throughout their diagnosis and treatment. Considerations for neighborhood safety and green space were discussed to have salutary impacts on health in addition to explorations about exposures to environmental pollutants due to proximity to industrial complexes. Education was described beyond the attainment of an academic degree and the inclusion of individual lived experiences to support the survivorship journey. SDOH remains an important consideration for QOL and survivorship, but the inclusion of these assessments and the implementation of solutions once needs are identified remain a challenge as the primary objective of the healthcare team is to treat the disease.

## Data availability statement

The raw data supporting the conclusions of this article will be made available by the authors, without undue reservation.

## Ethics statement

The studies involving humans were approved by Beckman Research Institute, City of Hope. The studies were conducted in accordance with the local legislation and institutional requirements. The participants provided their written informed consent to participate in this study.

## Author contributions

DT: Conceptualization, Data curation, Formal analysis, Funding acquisition, Investigation, Methodology, Project administration, Validation, Visualization, Writing – original draft, Writing – review & editing. BF: Formal analysis, Writing – review & editing. OO: Formal analysis, Writing – original draft, Writing – review & editing. AD: Formal analysis, Writing – original draft. LE: Methodology, Writing – review & editing. SM: Methodology, Writing – review & editing. DR: Writing – review & editing. RK: Writing – review & editing. JK: Conceptualization, Data curation, Formal analysis, Funding acquisition, Investigation, Methodology, Project administration, Resources, Supervision, Validation, Visualization, Writing – original draft, Writing – review & editing. VS: Conceptualization, Data curation, Formal analysis, Funding acquisition, Investigation, Methodology, Project administration, Resources, Supervision, Validation, Visualization, Writing – original draft, Writing – review & editing.
