# Supplementary material for: Social determinants of health and lung cancer surgery: a qualitative study
Source: Front Public Health. 2023 Oct 31;11:1285419. doi: 10.3389/fpubh.2023.1285419 (PMC10644827; doi:10.3389/fpubh.2023.1285419)
Supplement: Supplementary file 1 [file Data_Sheet_1.docx]

**Appendix A**

**Phase 1**: Share a video about the tale of two zip codes: <https://www.youtube.com/watch?v=Eu7d0BMRt0o>

- Have you ever heard of this concept that your zip code is more important than your genetic code?
- What were your impressions of the video? What resonated with your current experiences?
- Which town most represents your current living situation A-Town or B-ville?

**Phase 2:** *Neighborhood and Built Environment: As described in the video, where you live has a major impact on your health and quality of life.*

1. How would you describe the community that you live in? Describe the safety of your community, your water, street, open air space, pollution, and park availability, etc.
2. In what way [if any] may your neighborhood/environment have affected your experience with lung cancer?
3. If available, would you be interested in learning more about environmental exposures in your community?
4. What kinds of environmental exposure information would you like to know about your community?
5. Do you feel environmental exposure information would be helpful in planning your healthcare/cancer care?

*Social and Community Context: We want to know how your relationships and interactions with your friends, co-workers and community impact your quality of life.*

1. How would you describe your life [or lived experience] with lung cancer?
2. How would you describe your social network?
3. How has the lung cancer diagnosis changed your relationship?
   1. with others family/community members
   2. with your caregiver
   3. with yourself
      1. How did you cope with the stressors of your lung cancer journey?
      2. Do you use or have you used religion or spirituality throughout your lung cancer journey?
         1. Please provide examples related to your physical, spiritual, or psychological health, if appropriate.
         2. Describe your religious and/or spiritual background.
         3. When did you use religion or spirituality?
         4. How important was this usage in your journey?

*Health care Access and Quality: Health care access services can depend on several socioeconomic factors. For example, 1 in 10 people do not have health insurance or access to the health care services they need.*

1. Can you describe your relationship with the health care system during your lung cancer journey?
   1. How were you treated by your care team throughout your lung cancer journey?
   2. *Do you feel that your care team sees you as a person or only a patient?*
2. How would you describe your health care access and the quality of resources that you received during your cancer journey?
3. Did you receive all the services you needed during your diagnosis and after treatment?
4. Which services were missing? (e.g., psychosocial support services)
5. Palliative care services focus on providing relief from symptoms and stress of an illness. The goal is to improve your quality of life. Prior to this conversation, did you know about palliative care services at City of Hope?
   1. Did your provider discuss palliative care services with you?
   2. Did you have access to palliative care services?
   3. If you had access, did you receive palliative care services?
6. How far away was your provider?
7. Do you think this is a reasonable distance to travel for care?

*Education: Research tells us people with higher levels of education live healthier and longer lives.*

1. What are your thoughts about this statement?
2. What is the highest level of education of any type that you have completed?
3. Do you think your educational background supported your lung cancer journey?
   1. *How did your educational background affect your lung cancer care?*
4. Are there additional questions we should be asking to determine how much education someone has that would help them with their cancer journey?

*Economic Stability: This domain focuses on your economic mobility, food security, employment, and housing.*

1. Based on the definition you just read, do you have any unmet economic concerns that you need support with?
2. What are those concerns?
3. How would meeting these concerns improve your overall quality of life?
4. Would addressing these concerns also improve the quality of life of your caregiver? Why or why not?
5. How could the care team have supported you during this time?
6. In the last 12 months, have you worried whether your food would run out before you got money to buy more?
7. In the past 12 months have you felt, the food that you bought just didn’t last and you didn’t have money to get more?
8. Has your economic condition affected your experience with lung cancer? If yes, how?
9. Has lung cancer impacted your economic condition? If yes, how?

**Phase 3:** *Before you go, please answer these last two questions to let us know if we missed any information about your journey.*

1. Do you feel that social determinants of health information, like the ones you shared with us today about your *environment, your social network including coping, education*, should be included as part of your treatment plan discussions with your cancer care team?
2. How should this information be used in your treatment care plan?

That concludes our interview. Thank you so much for coming and sharing your thoughts and opinions with us. If you have additional information that you did not get to say during the interview, please feel free to email me at [teteh@chapman.edu](mailto:teteh@chapman.edu).
